# Supplementary material for: Evaluation of the Adverse Effects of Chronic Exposure to Donepezil (An Acetylcholinesterase Inhibitor) in Adult Zebrafish by Behavioral and Biochemical Assessments
Source: Biomolecules. 2020 Sep 18;10(9):1340. doi: 10.3390/biom10091340 (PMC7564914; doi:10.3390/biom10091340)
Supplement: Supplementary file 1 [file biomolecules-10-01340-s001.pdf]

Supplementary Materials

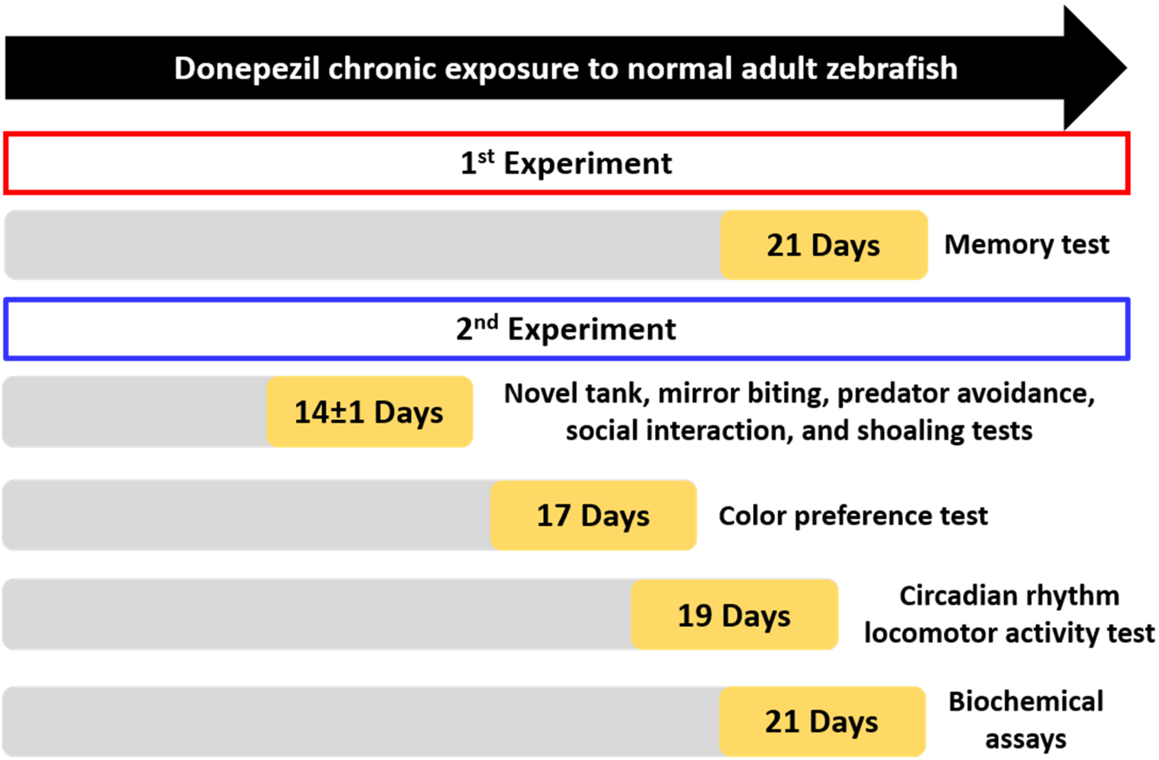

Figure S1. Schematic diagram of the evaluation of the neurobehavioral toxicity of donepezil in normal adult zebrafish. The yellow color indicates the time points (days) for conducting either behavioral or biochemical assays.

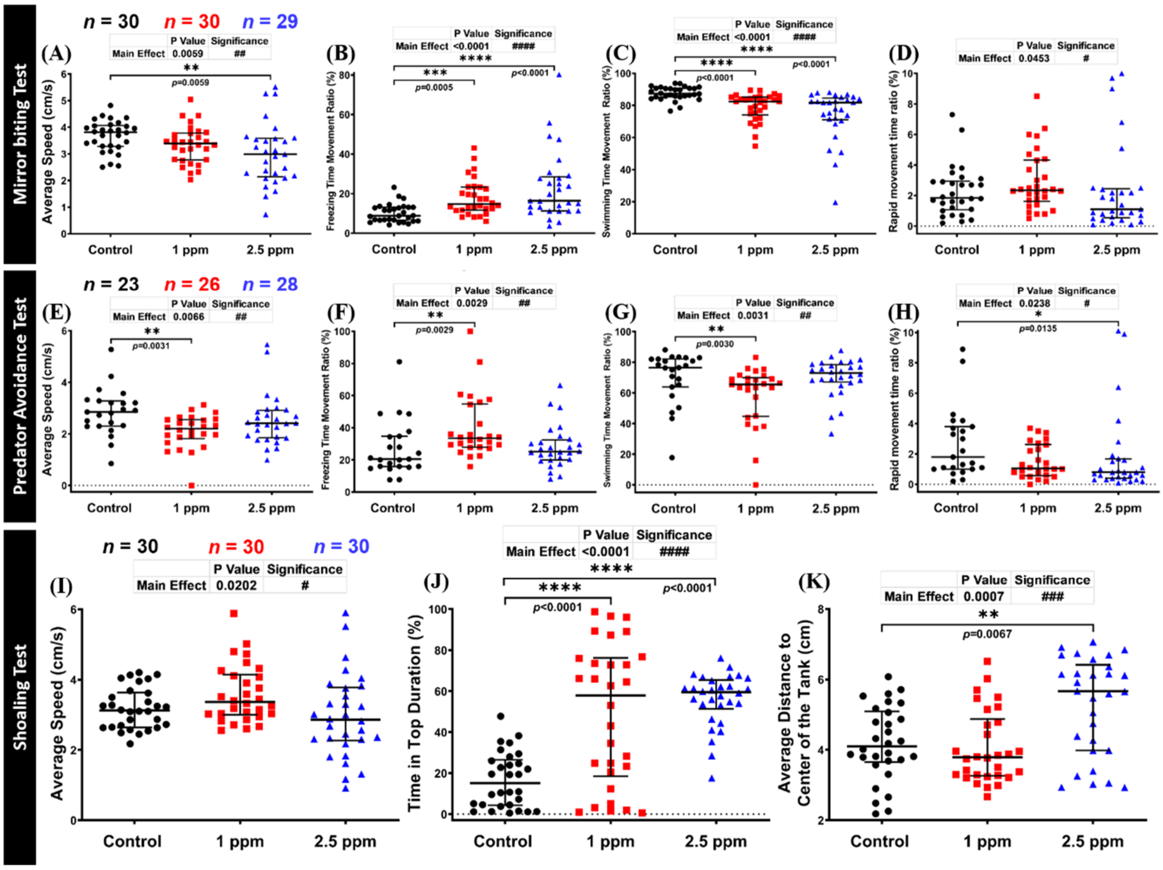

Figure S2. Mirror biting, predator avoidance, and shoaling behavior endpoint comparisons between the control, 1 ppm, and 2.5 ppm donepezil-exposed zebrafish groups after 14 days of exposure: (**A**, **E**) average speed; (**B**, **F**) freezing time movement ratio; (**C**, **G**) swimming time movement ratio; and (**D**, **H**) rapid movement time ratio were analyzed in the mirror biting and predator avoidance tests, respectively. (**I**) Average speed; (**J**) time in top duration; and (**K**) average distance to the center of the tank were analyzed in the shoaling test. *N* number for each group in each test is highlighted in (**A**), (**E**), and (**I**). The data are expressed as the median with interquartile range and were analyzed using the Kruskal–Wallis test followed by Dunn's multiple comparisons test (#/* $p < 0.05$, ##/** $p < 0.01$, ###/*** $p < 0.005$, ####/**** $p < 0.001$).

Table S1. The n number value calculated with 90% confidence interval (Z = 1.645) and a margin of error of 7 units (E = 7) from each behavior test endpoint in control fish.

Zebrafish Behavior Tests	Zebrafish Behavior Endpoints		Standard Deviation/σ	n Number $n = (\frac{Z\sigma}{E})^2$
**Novel Tank Test**	Average Speed		0.88	0.04
	Freezing Time Movement Ratio		14.01	10.83
	Time in Top Duration		29.85	49.20
	Number of Entries to The Top		2.04	0.23
	Latency to Enter The Top		21.41	25.31
	Total Distance Traveled in The Top		54.65	164.92
**Mirror Biting Test**	Mirror Biting Time Percentage		13.22	9.65
	Longest Duration in The Mirror Side		4.84	1.30
	Average Speed		0.58	0.02
	Freezing Time Movement Ratio		4.60	1.17
	Swimming Time Movement Ratio		4.14	0.94
	Rapid Time Movement Ratio		1.63	0.15
**Predator Avoidance Test**	Predator Approaching Time		15.05	12.51
	Average Distance to Separator		2.06	0.23
	Average Speed		0.91	0.05
	Freezing Time Movement Ratio		17.10	16.15
	Swimming Time Movement Ratio		16.97	15.90
	Rapid Time Movement Ratio		2.30	0.29
**Social Interaction Test**	Interaction Time Percentage		24.27	32.53
	Average Distance to Separator		1.69	0.16
	Longest Duration in Separator Side		9.03	4.50
**Shoaling Test**	Average Inter-fish Distance		0.95	0.05
	Average Nearest Neighbor Distance		0.63	0.02
	Average Farthest Neighbor Distance		1.03	0.06
	Average Speed		0.58	0.02
	Time in Top Duration		13.39	9.90
	Average Distance to Center of the Tank		1.06	0.06
**Circadian Locomotor Activity**	Average Speed	Light Cycle	3.37	0.63
		Dark Cycle	0.73	0.03
	Average Angular velocity	Light Cycle	1.72	0.16
		Dark Cycle	1.20	0.08
	Meandering	Light Cycle	74.97	310.39
		Dark Cycle	81.74	368.98
	Freezing Time Movement Ratio	Light Cycle	22.10	26.97
		Dark Cycle	18.30	18.49
	Swimming Time Movement Ratio	Light Cycle	17.79	17.48
		Dark Cycle	17.80	17.50
	Rapid Time Movement Ratio	Light Cycle	16.16	14.42
		Dark Cycle	1.10	0.07
**Color Preference Test**	Green–Blue		0.20	0.00
	Green–Yellow		0.20	0.00
	Red–Blue		0.11	0.00
	Red–Yellow		0.20	0.00
	Green–Red		0.49	0.01
	Blue–Yellow		0.17	0.00
	**AVERAGE**			**25.14**

Table S2. The P value from main effects of each circadian locomotor activity endpoint after Kruskal–Wallis test (# $p < 0.05$, #### $p < 0.001$).

	Circadian Locomotor Activity Endpoints	*p* Value	Significance
Day Cycle	Average Speed	<0.0001	####
	Average Angular Velocity	0.0162	#
	Meandering	<0.0001	####
	Freezing Movement Time Ratio	<0.0001	####
	Swimming Movement Time Ratio	<0.0001	####
	Rapid Movement Time Ratio	<0.0001	####
Night Cycle	Average Speed	<0.0001	####
	Average Angular Velocity	0.3534	ns
	Meandering	<0.0001	####
	Freezing Movement Time Ratio	<0.0001	####
	Swimming Movement Time Ratio	<0.0001	####
	Rapid Movement Time Ratio	<0.0001	####
